# Semi-Quantitative Assessment of Surgical Navigation Accuracy During Endoscopic Sinus Surgery in a Real-World Environment

**DOI:** 10.1177/00034894241286982

**Published:** 2024-10-01

**Authors:** David Z. Allen, Jason Talmadge, Martin J. Citardi

**Affiliations:** 1 Department of Otorhinolaryngology—Head and Neck Surgery, McGovern Medical School, The University of Texas Health Science Center at Houston, Houston, TX, USA; 2 ENT Specialists PC (Omaha, NE), Omaha, NE, USA

**Keywords:** sinus navigation, endoscopic sinus surgery, image guided surgery

## Abstract

**Introduction:**

Although surgical navigation is commonly used in rhinologic surgery, data on real world performance are sparse because of difficulties in collecting measurements for target registration error (TRE). Despite publications showing submillimeter TRE, surgeons do report TRE of >3 mm. We describe a novel method for assessing TRE during surgery and report findings with this technique.

**Methods:**

The TruDi navigation system (Acclarent, Irving, CA) was registered using a contour-based protocol. The surgeon estimated target registration error (e-TRE) at up to 8 points (anatomic regions of interest [ROI]) during endoscopic sinus surgery (ESS). System logs were used to simulate the localization for quantitative assessment of TRE (q-TRE).

**Results:**

We performed 98 localizations in 20 patients. The ROI in the sinuses were ethmoid (33 sites), maxillary (28 sites), frontal (17 sites), and sphenoid (22 sites). For localizations, mean qTRE and eTRE were 0.93 and 0.84 mm (*P* = .56). Notably, 80% of qTRE and 81% of eTRE were 1 mm or less. Mean qTRE and eTRE were less for attending-performed registrations at the maxillary, frontal and sphenoid.

**Conclusion:**

Surgical navigation accuracy, as measured by qTRE and eTRE, approaches 1 mm or better at all sinus sites in a real-world setting for 80% of localizations. The qTRE method provides a unique approach for assessing TRE. Surgeons underestimate TRE (overstate navigation accuracy), but this difference does not seem to be statistically significant. Registration performed by trainees yields higher TRE than registration performed by attendings. These data may be used to guide navigation optimization.

## Introduction

Using surgical navigation (SN), rhinologic surgeons can directly localize points in the operating field against the preoperative computed tomography (CT) scan. Such technology has been instrumental in the growth of endoscopic sinus surgery (ESS) from limited procedures of the anterior ethmoid and maxillary sinuses to complex procedures encompassing the entire anterior skull base and has seen remarkable improvements over the past 2 decades.^1^ SN has been shown to increase surgeon comfort during ESS while reducing both minor and major complications.^2-5^ Despite relatively wide acceptance of SN, some surgeons express frustrations with apparent inaccuracies of SN.

To better understand the limitations of SN, registration error (RE) theory provides a useful framework. Target registration error (TRE) is the distance between a point within the surgical field and the measured position within the imaging space defined by the preoperative CT scan. After registration, surgeons will estimate TRE by localizing known anatomical landmarks to assess the intra-operative accuracy of the registration.^6^ Reported TRE values are often near 2 mm, but some systems report errors of less than 1 mm.^7,8^ In these reports, the methodology for calculating TRE is sparsely described, and such methods often are not applicable to a real-world environment. Furthermore, many surgeons anecdotally report TRE values over 3 mm that they deem unacceptable.

In this report, we describe a novel method for determining TRE at various subsites during real-world ESS. This information fills an important gap in understanding the limitations and potential for SN.

## Methods

All cases were image-guided functional endoscopic sinus surgery procedures performed with the TruDi surgical navigation system version 2.3 (Acclarent, Irvine, CA) throughout the months of August-December 2022. Data were obtained from consecutive cases in which 2 or more subsites are accessible for determining TRE. A contour-based registration was performed by an attending surgeon or trainee (resident or fellow) in accordance with the device’s instructions for use. Images were saved to a drive and reviewed at a separate time. During surgery, the surgeon recorded snapshots of localizations in designated regions of interest (frontal sinus, ethmoid sinus, maxillary sinus, and sphenoid sinus). Selection of regions of interest for each case was dependent upon the extent of the surgery; each sinus that was operated upon was designated a region of interest for each case. In addition, the surgeon selected each snapshot so that the instrument tip was resting against at least 1 structure (ie, medial orbital wall) or preferably 2 or 3 structures (ie, medial orbital wall and adjacent residual ethmoid partition). Through point selection, the surgeon chose representative localizations for system accuracy at that region of interest. These snapshots were later reviewed to determine estimated TRE (eTRE) and quantitative TRE (qTRE) for each localization.

For eTRE measurement, a single attending surgeon estimated the error by reviewing the snapshot images later (not during the actual surgical procedure). Each snapshot included the localization on the orthogonal CT images (axial, coronal, and sagittal images), plus a representation of the instrument used for localization. Importantly, TruDi system records on each snapshot the marker icon for probe to scale, and each instrument (probe vs wire vs suction) has unique icon. As a result, the instrument itself, as represented on the recorded snapshots, represents an internal standard for visual estimates of distance. For instance, the outer diameter of the probe is 1.4 mm, the icon for the probe can be used as an internal standard of 1.4 mm for estimating distances. Furthermore, the geometry of the probe tip also influences determination of TRE. The probe provides a more precise tool for localization since its outer diameter is only 1.4 mm (vs 3.4 mm for the TruDi system’s suction). In addition, it is important to remember that TruDi system tracks the center of the instrument tip. For eTRE, the surgeon used the known geometry (including the size of the instrument tip) of the surgical tool as an informal calibration metric for estimating distances through direct inspection of digital images at a computer workstation. In short, eTRE is a visual estimate, obtained after the surgery and based upon the instrument’s unique visual representation, which served an internal “yardstick.”

**Figure 1.**
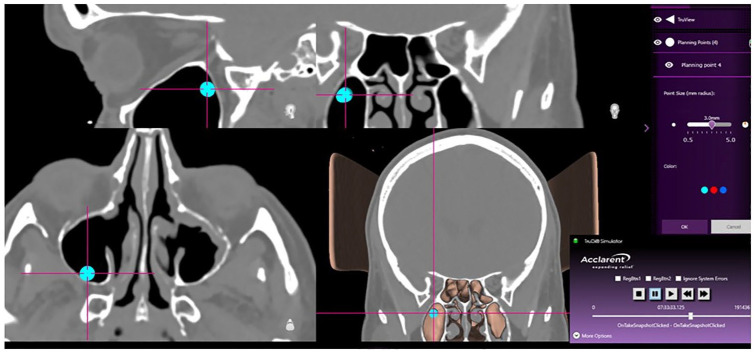
For the qTRE measurement, a planning point was placed at the localization crosshairs and then enlarged until it touched the adjacent bony structure. The measurement was derived from the radius of the planning point.

The qTRE measurement process relied upon 2 features of the TruDi navigation system. First, TruDi records a comprehensive log of all events during every procedure. This log is intended for system troubleshooting and can be exported at the end of each case. In practice, the log is a recording of all activity on TruDi during a s case. Secondly, TruDi planning software includes planning points, whose size can be set in increments of 0.5 mm. The planning points can be used for visual estimates of distance. For the qTRE measurement, the snapshots were used to determine the time point in the system case logs, and then TruDi tools were used to measure the distance from the localization point to the nearest anatomic structures. The software tools for displaying the system logs are not commercially available; the engineering team at Biosense Webster provided these data. Planning points of known sizes were placed at the localization point, and qTRE measurement was defined as the radius of the planning point that touched the adjacent anatomic structures (Figure 1). If the adjacent structure was between 2 increments, the user selected the lower increment if the difference was less an 0.25 mm or the higher increment if the distance was greater than 0.25 mm; in practice, this was a visual estimate. All measurements were quantified in 0.5 mm increments. This was chosen because the planning points are sized in 0.5 mm increments.

Comparative analytics were performed by using Welch’s *t*-tests, Wilcoxon-Mann *t*-tests, chi-squared tests, and ANOVA using R studio.

The Institutional Review Board of The University of Texas Health Sciences Center at Houston reviewed and approved the project.

## Results

A total of 98 localizations in 20 patients were performed. Attendings performed most of the registrations (82%), followed by fellow and resident (10% and 8%, grouped as “Trainees”). The regions of interest (ROI) were ethmoid sinus (33 sites), maxillary sinus (28 sites), frontal sinus (17 sites), and sphenoid sinus (22 sites). The surgical tool utilized for localization was most often the probe (83%) followed by curette (11%), and lastly Fraser suction (6%).

An average of 276 points were collected for each registration, with a standard deviation of 60. While trainees had greater mean number of points collected for registration, the difference was not statistically significant (266 vs 315, *P* > .05).

**Figure 2.**
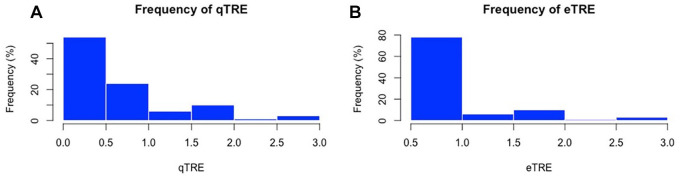
Histogram plots depicting the frequency (%) of eTRE (A) and qTRE (B).

**Figure 3.**
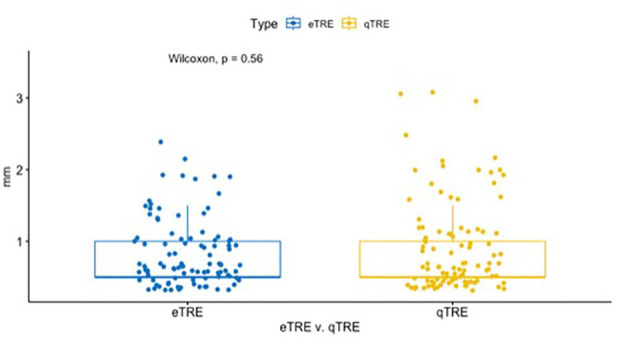
Layered box plots comparing the qTRE vs eTRE.

Mean qTRE and eTRE were 0.93 ± 0.64 and 0.84 ± 0.49 mm respectively (*P* = .56, Wilcoxon rank sum). Notably 80% of qTRE values and 81% of eTRE values were 1 mm or less (Figure 2A and B). The percentage of TRE values below 1 mm were similar at each subsite (Table 1). The distribution of qTRE and eTRE values were similar (Figure 3). Interestingly, the frontal sinus had the highest percentage of qTRE under 1 mm; however, the differences among ROI was not statistically significant (*P* > .05, Fisher Test). The ethmoid sinus had the highest percentage of eTRE under 1 mm; however, the difference between ROI was not statistically significant (*P* > .05, Fisher Test).

**Table 1.** Percentage of eTRE and qTRE Values Less Than 1 mm by Subsite.

ROI	eTRE (%)	qTRE(%)
Maxillary	73	69
Ethmoid	85	88
Frontal	88	83
Sphenoid	77	77
All sites	81	80

**Figure 4.**
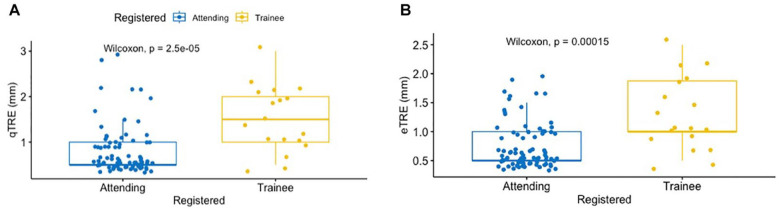
Layered boxplots comparing the qTRE (A) and eTRE (B) between attending surgeon and trainee.

Mean qTRE and eTRE was significantly lower in attending-performed registrations compared to trainee-performed registration with values of 0.81 mm (0.5-2) and 0.75 mm (0.5-2) vs 1.5 mm (0.5-2) and 1.27 mm (0.5-3) respectively (*P* < .01; Figure 4A and B). Mean eTRE was less for attending-performed registrations at all ROI subsites, but the difference was only significant at the maxillary, frontal, and sphenoid sinuses (Table 2). Mean qTRE was less for attending-performed registrations at all ROI subsites, but the difference was only significant at the maxillary and frontal sinuses (Table 3).

**Table 2.** Mean eTRE Values by Region of Interest for Attending Surgeon Versus Trainee.

ROI	Overall	Attending (n = 78)	Trainee (n = 20)	*P*
Maxillary	0.88	0.76	1.4	.02
Ethmoid	0.79	0.73	1.0	.36
Frontal	0.76	0.67	1.5	.03
Sphenoid	0.96	0.83	1.5	.04
All sites	0.85	0.75	1.27	<.001

*Note*. Wilcoxon Mann test was used to calculate *P*-values.

**Table 3.** Mean qTRE Values by Region of Interest for Attending Surgeon Versus Trainee.

ROI	Overall	Attending (n = 78)	Trainee (n = 20)	*P*
Maxillary	1.1	0.93	1.8	<.01
Ethmoid	0.83	0.77	1.1	.11
Frontal	0.79	0.70	1.5	.03
Sphenoid	0.98	0.81	1.8	.07
All sites	0.93	0.81	1.5	<.001

*Note*. Wilcoxon Mann test was used to calculate *P*-values.

**Figure 5.**
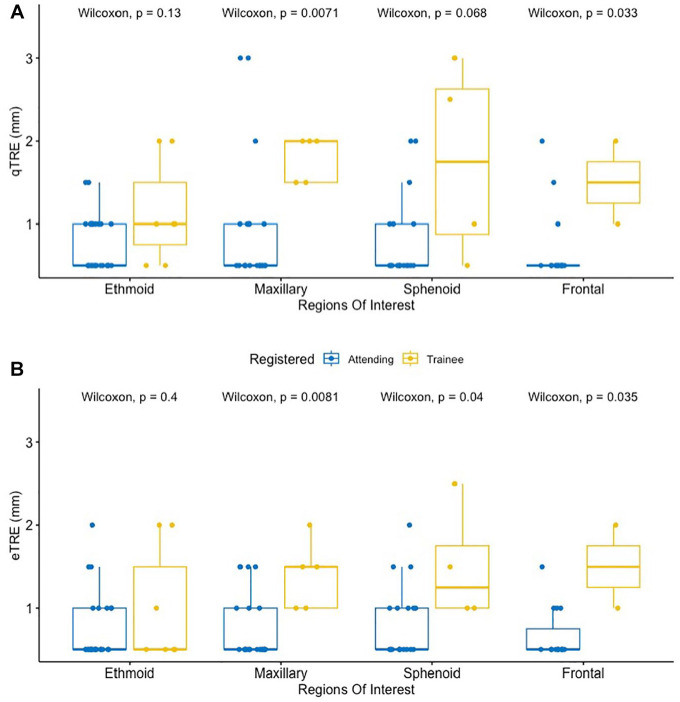
Multi-layered boxplots depicting the difference in qTRE (A) and eTRE (B) for attending-performed vs trainee-performed registration by ROI. *Note*. Only qTRE had a statistically significant variance when comparing attending vs trainee (*P* = .01, *F*-test).

The distribution of TRE values for attending- versus trainee-performed registration also differed as TRE values fell in a narrower range for attending-performed registration (Figure 5A and B); however just qTRE had a statistically significant variance when comparing attending versus trainee (*P* = .01, *F*-test).

## Discussion

In this report, we describe a novel system for assessing TRE during real world ESS. Prior reports have given accuracy measurements, but the details of the methods in these reports are sparse. In some instances, the reported “accuracy value” represents other measures of the registration process, but not true TRE. The methods described here provide robust TRE values of SN performance at various subsites during ESS. This technique is a useful framework for future studies of navigation systems in “real-world” settings.

In this investigation, we performed the first real-world quantitative assessment of TRE in IG-FESS that has been reported for publication. This was done through an analysis of qTRE (radius of the planning point that touched the adjacent anatomic structures) and eTRE (surgeon estimation of the discrepancy). Overall eTRE was 0.85 mm (0.5-2.5 mm) and overall qTRE was 0.93 mm (0.5-3 mm), with 81% of eTRE values and 80% qTRE values less than 1 mm. Performance at subsites was comparable throughout. This level of performance exceeds the expectations of most surgeons. These data confirm the remarkable robustness of the registration protocol and overall system performance in a real-world operating room setting.

Despite this level of overall importance, some localizations had TRE values above the clinically acceptable level of 1.5 mm or better. This study did not specifically examine these occurrences. Reasons include random perturbation of the electromagnetic fields used for sensing instrument position, human errors, and hardware failures. It is not possible to exclude registration algorithm errors that calculate an inaccurate registration because of CT scan factors, point distribution features, etc. Notably, the use of instruments with distal sensors eliminates errors due to probe flexing.

When comparing between attending and trainee registration, attendings had superior mean qTRE and eTRE by approximately 0.5 mm. This suggests that attending surgeons achieve lower TRE—a finding that is consistent with prior reports.^9,10^ It should be emphasized that performance of the registration step was independent of the assessment of eTRE and qTRE, and that information about the person performing the registration was not immediately available at the time of assessing eTRE and qTRE. Moreover, mean eTRE was less for attending-performed registrations at all ROI subsites, but significantly different at the maxillary, frontal, and sphenoid sinuses (Table 2). Further, mean qTRE was less for attending-performed registrations at all ROI subsites, but the difference was only significant at the maxillary and frontal sinuses (Table 3). The reason for these discrepancies is not clear from the current data; however, one may surmise that registration is a skill that improves with practice. Considering the consistent pattern observed here and in prior publications, adequate resident supervision and training are important in training programs. Specific education for registration is encouraged.

Trainees also tended to use more points in their registrations and yet had higher TRE values. This is consistent with the observation that greater numbers of registration points alone are sufficient for achieving an ideal TRE. Point acquisition techniques, including avoiding deformation of skin contours, are clearly important during the registration process. During point acquisition, probe orientation to maintain contact with the surface also seems to influence TRE. Prior work has shown that point distribution, as well as surgeon experience, impact TRE.^10^

Interestingly, surgeon-performed eTRE was 0.84 mm, and independently derived qTRE was 0.93 mm. Although this difference is not clinically meaningful, it suggests that surgeons may be inclined to overestimate the reliability of SN. The caveat that surgeons should consistently verify TRE throughout surgery remains true even on the latest generation of technology.

TRE is a vector in that it has x-direction, y-direction, and z-direction components. In clinical practice, surgeons may observe differences in apparent TRE in each of these directions. Ideally, TRE measurements should capture xyz values separately, but in practice doing so is difficult due to technical limitations in navigation software. For the current report, we report overall TRE values, not separate values for x-direction, y-direction, and z-direction. TruDi simply could not provide the vector data. Measurements for qTRE and eTRE were both obtained as single values, without regard to assessing x-, y-, and z- directions independently. In theory, a measurement could be low in two cardinal directions and higher in the third cardinal direction. Fortunately, this phenomenon is not common. For the purposes of this study, both qTRE and eTRE were quantified on an overall, or “global” scale; that is, in the event of a discrepancy of an estimate in one of the cardinal directions, the reviewer assigned a composite value. The values were also aggregated in 0.5 mm increments, in part because of the restrictions of the sizing targets in the TruDi software. In addition, the use of 0.5 mm increments approximated the slice thickness of the CT scans (all of which were 0.5 mm thickness or less).

The methodology for qTRE is also noteworthy, because it is not merely a visual estimation performed by the surgeon. The qTRE technique relies upon system logs to simulate navigation during the actual surgery. To our knowledge, this approach relies upon software event logging that is proprietary to the TruDi system. It is possible that other navigation systems create similar case logs which are not available to surgeons. Regardless, such case logs are important for validation of accuracy, and we believe that this approach should be encouraged for future reports on registration accuracy.

The visual estimates performed for eTRE are more robust than prior reports. The eTRE assessments were performed from screen captures that included an icon for the instrument tip. Because the TruDi display scales the icon to the actual size of the instrument tip, the icon served an informal “measuring stick.” In addition, the estimates based upon the icon also allowed the surgeon to compensate for the instrument thickness.

It should be noted that most localizations were performed with a tracked fine tip probe, rather than a suction. A probe with a smaller outer diameter allows more precise localizations since the probe tip more easily contacts small spaces in the paranasal sinuses.

This investigation carries potentially significant limitations. First, this was retrospective in nature and as such, data collection could be subject to sampling bias. However, there was no recall bias associated with this study as the variables of interest were collected at the time of surgery and data are objective in nature. Furthermore, qTRE measurements were performed independently. This study also had a small number of patients within the “Trainee” group, thus limited the external validity. Given that trainees can be at any point in their training, this could lead to significant variability within their outcomes. There were also large standard deviations in the analysis, suggestive of significant variance, even though we did find statistical significance. Importantly, our investigation only included senior residents or fellows and both the eTRE and qTRE followed normal distributions (Shapiro-wilk test > .05) suggesting that there was not significant skew that could alter comparative analysis. Lastly, the current study only examined the performance of a specific system at a single institution. While it would be feasible to assess this system at other institutions using similar methodology, the absence of certain features on competitor navigation system precludes the use of the current methodology with those systems, unless the software packages in those systems were significantly modified.

## Conclusion

Surgical navigation accuracy, as measured by qTRE and eTRE, approaches 1 mm or better at all sinus sites in a real-world setting for approximately 80% of all localizations. The qTRE method provides a robust approach for assessing TRE. Surgeons tend to underestimate TRE (overstate navigation accuracy), but this difference was not statistically significant. Registration performed by trainees yields a higher TRE than registration performed by attending surgeons. These data may be used to guide navigation system optimization and design future studies in real-world settings.
